# The impact of rainfall changes on soil bacterial and fungal communities in *Pinus yunnanensis*

**DOI:** 10.3389/fmicb.2025.1613698

**Published:** 2025-07-03

**Authors:** Lin Wang, Qiong Dong, Shaojie Zheng, Xingze Li, Huiping Zeng, Yu Chen, Shunrou Shi

**Affiliations:** ^1^College of Forestry, Southwest Forestry University, Kunming, China; ^2^Key Laboratory of Southwest Biodiversity Conservation, State Forestry and Grassland Administration, Kunming, China

**Keywords:** *Pinus yunnanensis*, changes in rainfall, microbial community, functional composition, antioxidant enzyme system

## Abstract

**Introduction:**

To explore the differences in soil bacterial and fungal community characteristics under *Pinus yunnanensis* forests with different rainfall amounts, natural *Pinus yunnanensis* forests in Guangnan County (GN), Jianshui County (JS), and Yuanjiang County (YJ) of Yunnan Province, China, were selected as the research objects.

**Methods:**

Three 20 × 20 standard plots were set up for each forest stand. The antioxidant system of *Pinus yunnanensis* seedlings in different plots was determined, and high-throughput sequencing technology was used to analyze soil bacteria and fungi.

**Results:**

The results showed that during the growth process of *Pinus yunnanensis*, the activities of SOD and POD, as well as the content of Pro, were highest in GN, while the content of SP was highest in the YJ. Significant differences were found in the *α*-diversity of soil bacterial and fungal communities among regions (*p* < 0.05). Specifically, for soil bacteria, the Shannon, Chao1, and ACE (Abundance-based Coverage Estimator) indices were highest in GN and lowest in JS; for fungi, the Chao1 and ACE indices were highest in YJ and lowest in JS. The dominant phyla of soil bacterial communities in GN and JS regions were *Acidobacteria, Proteobacteria*, and *Chloroflexia*, whereas in the YJ region were *Acidobacteria, Verrucomicrobiota*, and *Proteobacteria*. The dominant phyla of soil fungal communities across all regions were *Basidiomycota* and *Ascomycota*. Through the comparison of Tax4Fun and FUNGuild functions, it was found that more than 80% of the main functions of soil bacteria in various regions are related to metabolism. Soil fungi were primarily of the *Symbiotroph* nutritional functional types, and the main functional group was *Ectomycorrhizal* fungi. In addition, changes in rainfall had minimal effect on the functional abundance of soil bacterial communities but had a more significant impact on the functional abundance of fungal communities.

**Discussion:**

In summary, the soil bacterial community diversity and richness index of *Pinus yunnanensis* in the GN region is the highest, and the bacterial phylum related to plant growth and *Symbiotroph* fungi account for the highest proportion. This study elucidated the response mechanism of *Pinus yunnanensis* to changes in rainfall from a microbial perspective, providing a theoretical basis for their growth being regulated by rainfall.

## 1 Introduction

The IPCC Sixth Assessment Report clearly states that anthropogenic CO₂ emissions are causing global ecosystem warming. With global warming intensifying the hydrological cycle, global average precipitation is projected to increase by 1–3% for every 1°C rise in global mean temperature (IPCC, 2023). The rainfall patterns in different regions, including rainfall amount, intensity, and seasonal rainfall distribution, have also changed. The terrain in southwestern China is complex, comprising plateaus, mountains, and other features that make up the significant landforms of the continent (Zhang et al., 2014), causing spatial imbalances in precipitation across the region. Yunnan is located on a low-latitude plateau within the subtropical monsoon zone. Due to its unique geographical environment and climatic conditions, precipitation varies significantly across different areas. Due to the dual influence of the southwest monsoon and terrain, the annual rainfall in Xishuangbanna, southwestern Yunnan, can reach 1,500–2,500 mm (Wang et al., 2021). Southeast Yunnan is in a subtropical humid climate zone, characterized by abundant precipitation and an annual rainfall of 1,000–1,400 mm. In contrast, the precipitation in central Yunnan (e.g., Kunming, Yuxi) is relatively low, below 900 mm (Shi, 2018). However, in recent years, anomalous precipitation patterns have led to frequent rainstorms and droughts, significantly impacting regional vegetation. Excessive moisture reduces soil oxygen content, adversely affecting plant morphology and physiology, ultimately inhibiting normal growth. Liu et al. (2025) found that waterlogging stress inhibited height growth and reduced root and stem biomass accumulation in *Ochroma lagopus* seedlings. Conversely, drought stress can alter the allocation strategy of plant biomass (Yong et al., 2025) and restrict nutrient uptake, transport, and storage, ultimately leading to poor growth or even plant death (Wen et al., 2024). Zheng et al. (2025) found that a 40% reduction in rainfall inhibits the accumulation of biomass in various organs of *Cyphomandra betacea* seedlings. Similarly, Zong et al. (2025) found that all biomass metrics of *Xanthoceras sorbifolium* Bunge declined with increasing drought intensity, indicating hindered accumulation and growth of above- and below-ground biomass, consequently impairing overall plant growth.

The osmoregulatory substances mainly include proline (Pro) and soluble protein (SP), which are the main regulatory mechanisms for plants to respond to changes in rainfall (Xiong et al., 2023). Studies have shown that the Pro content in *Robinia pseudoacacia* Linn seedling leaves accumulates rapidly under severe drought stress, thereby maintaining the osmotic potential of leaves and other tissue cells (Ma et al., 2012). However, the SP and Pro content in *Pinus sylvestris* var. *mongolica* needles shows no significant correlation with increased rainfall but is negatively correlated with decreased rainfall (Chu et al., 2011). Under normal growth conditions, plants’ reactive oxygen species (ROS) maintain a dynamic balance. However, when subjected to abiotic stress such as drought, the ROS balance in plants is disrupted, leading to excessive accumulation. This accumulation can cause membrane lipid peroxidation and cell damage and, in severe cases, even lead to plant death (Fang and Xiong, 2015). To counteract this, plants scavenge ROS through the combined action of antioxidant enzymes, including Superoxide Dismutase (SOD) and Peroxidase (POD). SOD, a key enzyme for eliminating O₂^−^, catalyzes its dismutation into H₂O₂ and O₂. POD then further catalyzes the dismutation of H_2_O_2_ into H_2_O or other harmless products (Laxa et al., 2019). This coordinated action maintains ROS at low levels, ensuring normal plant physiological activities. Research has found that the POD activity in the leaves of *Reaumuria soongorica* seedlings decreases with increasing rainfall (Ji, 2017). In contrast, the SOD and POD activities of *Taxus cuspidata* seedlings showed a trend of first increasing and then decreasing with the intensification of drought stress (Li et al., 2025). These findings indicate that plant species exhibit significant differences in the content and dynamics of osmoregulatory substances and antioxidant enzyme activities in response to varying water conditions.

Soil microorganisms are essential driving forces for soil material transformation, nutrient cycling, and energy flow and are crucial for the stability of ecosystems (Zhang Z. L. et al., 2025). However, the frequent alternation of dry and wet soil moisture environments caused by rainfall changes significantly impacts the structure, composition, and activity of microbial communities, which in turn affects soil biogeochemical processes mediated by microorganisms (Zhang et al., 2020). Changes in rainfall can significantly affect soil moisture availability, thereby regulating the growth and distribution of both aboveground plants and soil microorganisms (Knapp et al., 2017). This impact is mainly achieved through two mechanisms: first, water fluctuations directly alter the growth rates, population sizes, and community composition of microorganisms (Kim et al., 2012). Second, changes in rainfall can affect the dissolution and diffusion of nutrients by regulating soil moisture content, indirectly altering the structure and diversity of microbial communities. It is worth noting that soil microbial responses to water changes vary significantly across regions (Barnard et al., 2015), and some microorganisms sensitive to water potential changes may decline or even disappear (Sun et al., 2012). Wang et al. (2023) found in simulated precipitation experiments that reduced precipitation decreased soil bacterial and fungal richness while increased precipitation significantly enhanced fungal richness. Similarly, Wu et al. (2020) found that drought significantly reduced bacterial abundance and diversity. Notably, excessively high soil moisture content can also inhibit microbial diversity (Yan et al., 2017). In summary, the relationship between soil microorganisms and water is complex, and predictable patterns of soil microbial community changes under altered rainfall regimes remain unclear. Therefore, further exploration is needed.

*Pinus yunnanensis* is mainly distributed in the Yunnan-Guizhou Plateau and the southeast Qinghai-Tibet Plateau, and it is widely distributed in Yunnan. As a principal afforestation species in southwestern China, it is known for its strong adaptability and drought tolerance (Zhou et al., 2024). Rainfall changes induced by global climate change exhibit significant spatial heterogeneity. The combined effects of rainfall changes and geographical environment differences still lack systematic exploration of the growth, physiological, and ecological processes, as well as the structure and functional characteristics of soil microbial communities under the forests of *Pinus yunnanensis*. Existing research on *Pinus yunnanensis* mainly focuses on seedling drought resistance (Liu et al., 2024) or gene expression regulation (Zhou et al., 2025). However, it neglects the synergistic adaptation mechanism between *Pinus yunnanensis* natural forests and soil microorganisms under actual rainfall changes. Especially in mountainous forest ecosystems, the coupling effect mechanism between plant antioxidant enzyme systems and soil microbial communities still lacks comprehensive research. According to the rainfall distribution pattern in Yunnan Province, it is high in the east and west and low in the center. This study selected three regions with rainfall differences in eastern Yunnan, namely Guangnan (GN), with abundant rainfall, Jianshui (JS) with moderate rainfall, and Yuanjiang (YJ) with less rainfall. Based on natural *Pinus yunnanensis* forests in these regions as the research subject and employing a combination of field investigation and laboratory experiments, this research systematically investigated: (1) the response of soil microbial community structure to rainfall changes and (2) the interaction mechanisms between the *Pinus yunnanensis* antioxidant enzyme system (SOD, POD, Pro, etc.) and the soil microbial community. This study not only addresses the theoretical gap concerning “plant–soil microbe synergistic adaptation to rainfall gradients” in forest ecosystems but also provides theoretical support from a microbiological perspective for explaining the driving phenomenon of *Pinus yunnanensis* growth in response to rainfall variations.

## 2 Materials and methods

### 2.1 Situation of the research area and sample plot

The research areas are located in Guangnan County (GN), Yunnan Province, (104°31′ ~ 105°39′E, 23°29′ ~ 24°28′N), at an altitude of 420 m ~ 2035 m. It belongs to the subtropical plateau monsoon climate, with an average temperature of 18.6°C and an annual total precipitation of 1063.4 mm. Rainfall is concentrated in July and August each year, and the frost-free period is long. Jianshui County (JS) (102°33′ ~ 103°11′E，23°12′ ~ 24°10′N) is located in the southern part of Yunnan Province, belonging to the Honghe Hani and Yi Autonomous Prefecture, with an average elevation of 1,500 meters, and a predominantly monsoon climate of the southern subtropical plateau characterized by wet and dry rain and heat in the same season, with an average temperature of 19.8°C, average annual precipitation of 790.9 mm. Yuanjiang County (YJ) (101°39′ ~ 102°22′E, 23°18′ ~ 23°55′N) is located in the high altitude plateau, belongs to the tropical monsoon climate, with winter warmth and summer heat, distinctive features of dry and wet seasons, the average annual temperature of 24.1°C, the average temperature in winter is 25.5°C, the average annual rainfall is 543.9 mm, the altitude is 327 m ~ 2,580 m, and there is no frost all year round. The abbreviations used in the text are detailed in Table A1.

### 2.2 Experimental design and sample collection

#### 2.2.1 Sample plot setting

In August 2024, the natural forests of *Pinus yunnanensis* in GN, JS, and YJ counties were selected as the research objects. Three 20 m × 20 m standard plots were set up along the same altitude in the middle slope, with a 20 m distance difference between plots. A total of nine plots were set up in the three regions. Global position system (GPS) is used to determine coordinate information such as the sample site’s longitude, latitude, and altitude. The management measures for natural pine forests in Yunnan are extensive, with no history of fertilization, and the soil type is mainly red. Record the tree height, diameter at breast height, and other information on *Pinus yunnanensis* in each standard plot. The detailed information of the sampling site and the physical and chemical properties of the soil in the sampling site are shown in Tables 1, 2, respectively.

**Table 1:** The basic situation of the sample plot.

Sampling point	Number	Longitude and latitude	Annual average precipitation /mm	Altitude /m	Temperature /°C	Slope direction	Canopy density	Average tree height/m	Average diameter at breast /cm	Forest stand age /a	Soil Type	Number of plants
GN	1	105°01′E,24°07′N	1097.68	1,288	16.7	southeast	0.60	12.59	18.59	35	Red soil	33
	2	105°01′E,24°08′N		1,323		South	0.50	12.07	17.18	35	Red soil	37
	3	105°01′E,24°04′N		1,370		South	0.55	12.23	17.97	33	Red soil	39
JS	1	102°76′E,23°71′N	813.19	1,697	18.5	South	0.65	8.67	11.08	30	Red soil	131
	2	102°75′E,23°71′N		1700		Southwest	0.55	8.41	9.84	32	Red soil	144
	3	102°76′E,23°71′N		1,672		Southeast	0.56	7.70	11.39	31	Red soil	69
YJ	1	102°13′E,23°58′N	543.9	1,541	24.8	West	0.65	18.67	16.53	34	Red soil	30
	2	102°23′E,23°54′N		1,497		Southwest	0.65	17.05	10.09	34	Red soil	32
	3	102°18′E,23°54′N		1,362		West	0.50	15.55	12.08	36	Red soil	59

**Table 2:** Soil physical and chemical properties.

Characteristics of soil	GN	JS	YJ
Soil pH	5.02	4.88	5.35
Total soil nitrogen (TN) /g∙kg^−1^	2.95	2.63	2.73
Total soil phosphorus (TP)/g∙kg-1	0.61	0.60	0.47
Soil organic carbon (SOC)/g∙kg-1	18.17	20.58	20.93
Soil moisture content /%	21.32	15.62	14.08

#### 2.2.2 Sample collection

Before collecting soil samples, remove the upper layer of litter and humus layer, and use the five-point sampling method to collect the topsoil of 0–20 cm. Mix five soil samples from each standard plot evenly, obtaining 9 (3 × 3) soil samples. After removing gravel and visible roots from the soil, sieve through a 2-mm sieve and store immediately at −80°C for high-throughput sequencing.

Three *Pinus yunnanensis* were selected as standard trees in each standard plot, and their needles were collected as samples. The needles from the outer layer of the tree crown in the east, south, west, and north directions were selected for each standard tree and mixed to obtain a sample. One sample was taken from each standard tree, and 27 (3 × 3 × 3) duplicate samples were collected from nine plots. The samples were sealed and returned to the laboratory for low-temperature storage to determine antioxidant enzyme activity.

### 2.3 Determination of antioxidant enzymes

The nitroblue tetrazolium (NBT) method was used to determine the SOD activity (Zuo, 1995). The reaction system of xanthine and xanthine oxidase produces superoxide anion (O_2_^−^), which can reduce nitrogen blue tetrazole to generate blue formazan, with absorption at 560 nm; SOD can clear O_2_^−^, thereby inhibiting the formation of formazan. The darker the blue color of the reaction solution, the lower the SOD activity, and vice versa, the higher the activity. The experimental steps were carried out according to the instructions of the reagent kit (Comin Biotechnology, China; www.cominbio.com, accessed on 15 May 2020). Measure the absorbance values of the measuring tube and blank tube at 560 nm, denoted as Am and Ab, respectively. FW: fresh weight.


$$\mathrm{PI}\;(\text{percentage of inhibition})=\frac{\mathrm{Ab}560-\mathrm{Am}560}{\mathrm{Ab}560}\times 100\%$$

$$\mathrm{SOD}\;\text{activity}\;(U/g,\mathrm{FW})=11.4\times \frac{\mathrm{PI}}{0.1\times (1-\mathrm{PI})}$$

The guaiacol method was used to determine the POD activity (Hammerschmidt et al., 1982). POD catalyzes the oxidation of specific substrates with H_2_O_2_ and has a characteristic light absorption at 470 nm. The experimental steps were carried out according to the instructions of the reagent kit (Comin Biotechnology, China; www.cominbio.com, accessed on 15 May 2020). Record the absorbance value A1 at 470 nm for 1 min and A2 after 2 min.


$$\mathrm{POD}\;\text{activity}\;(U/g,\mathrm{FW})=\frac{2000\times (A2-A1)}{0.1}$$

The bicinchoninc acid (BCA) method was performed to determine the SP content. Under alkaline conditions, cysteine, cystine, tryptophan, tyrosine, and peptide bonds in proteins reduce Cu^2+^ to Cu^+^; two molecules of BCA bind to Cu^+^, producing a purple complex at 562 nm. The experimental steps were carried out according to the instructions of the reagent kit (Comin Biotechnology, China; www.cominbio.com, accessed on 15 May 2020). The absorbance values (A) of the blank tube, standard tube, and measuring tube were measured at 562 nm and recorded as Ab, As, and Am, respectively.


$$\mathrm{SP}\;\text{content}\;(\mathrm{mg}/g,\mathrm{FW})=\frac{0.5\times (\mathrm{Am}562-\mathrm{Ab}562)}{0.1\times (\mathrm{As}562-\mathrm{Ab}562)}$$

The sulfosalicylic acid (SSA) method was used to determine the Pro content. Extract Pro with sulfosalicylic acid (SA). After heating treatment, Pro reacts with an acidic ninhydrin solution to produce a red color. After extraction with toluene, measure the absorbance at 520 nm. Record the absorbance value as A520 at 520 nm.


$$\mathrm{Pro}\;\text{content}\;(\mathrm{\mu g}/g,\mathrm{FW})=\frac{19.2\times (A520+0.0021)}{0.1}$$

### 2.4 Extraction, PCR amplification, and sequencing of microbial DNA

The total DNA of the microbial community was extracted according to the instructions of the CretMagTM Power Soil DNA kit. The DNA extraction quality was detected by 1% agarose gel electrophoresis, and the DNA concentration and purity were measured by NanoDrop2000 (Thermo Scientific, USA).

Use pre-primer341F (5′-CCTAYGGGRBGCASCAG-3′) and post-primer 806R (5′-GACTACHVGGGTWTCTAAT-3′) to amplify the V3-V4 regions of the soil bacterial 16S rRNA gene; PCR (Polymerase Chain Reaction) amplification of soil fungal ITS region was performed using pre-primer ITS1F (5′- CTGGTCATTTAGGAGAGATA-3′) and post-primer ITS2R (5′- GCTGTGTTCATGATGC-3). PCR products from the same sample were mixed and recovered on a 2% agarose gel, purified using the AxyPrep DNA Gel Extraction Kit (Axygen Biosciences, Union City, CA, USA), detected by electrophoresis on a 2% agarose gel, and quantified by Quantus™ Fluorometer (Promega, USA). The recovered products were purified by 2% agarose gel electrophoresis, detected and quantified by Quantus™ Fluorometer (Promega, USA). Library construction was performed using the VAHTS® Universal Plus DNA Library Prep Kit for MGI V2 (Vazyme Biotech Co., Ltd, China) on purified PCR products. (1) linker link; (2) Use magnetic beads to screen and remove adapter self-connecting fragments; (3) Enrichment of library templates using PCR amplification; (4) Magnetic bead recycling of PCR products; and (5) PCR product cyclization. Sequencing is performed using BGI Corporation’s DNBSEQ-G99 PE300 platform (Guizhou Weierlai Detection Technology Co., Ltd.). After the raw data were obtained by sequencing, they were quality-controlled and spliced using FASTP (0.20.0) and FLASH (1.2.7). The sequences after quality-controlled splicing were clustered by operational taxonomic unit (OTU), and chimeras were excluded based on 97% similarity using UPARSE software (7.1). OTU species taxonomy was annotated using RDP classifier (Ribosomal Database Project, version 2.2; http://rdp.cme.msu.edu/) against Silva 16S rRNA (v138), and unite8.0 gene databases, with a confidence threshold of 70%, and community composition was counted for each sample at different species taxonomic levels. Bacterial and fungal functions were performed using Tax4Fun software and FUNGuild.

### 2.5 Data processing

The data were statistically analyzed using Excel (2019) and SPSS (25. 0) software, and the differences in antioxidant enzyme activities of *Pinus yunnanensis* in different regions were analyzed by one-way analysis of variance (one-way ANOVA). Multiple comparisons of LSD were performed at the level of *p* = 0.05 to test the significance of the differences between different regions. Use Mothur (1.30.2) software to calculate soil bacterial and fungal alpha diversity indices (Shannon, Simpson, Ace, Chao1, etc.); Perform PCoA analysis and ANOSIM test using Bray–Curtis distance algorithm to evaluate the differences in bacterial and fungal structures between different regions. Using Kruskal–Wallis to select different species from various regions. Venn diagrams, bar plots, and PCoA were executed on the CloudTutu Bioinformatics Platform.^1^ Pearson correlation analysis investigated the correlation and significance between soil microbial communities and antioxidant enzyme systems.

## 3 Analysis of results

### 3.1 Effects of different rainfall on antioxidant enzyme activities of *Pinus yunnanensis*

**Figure 1:**
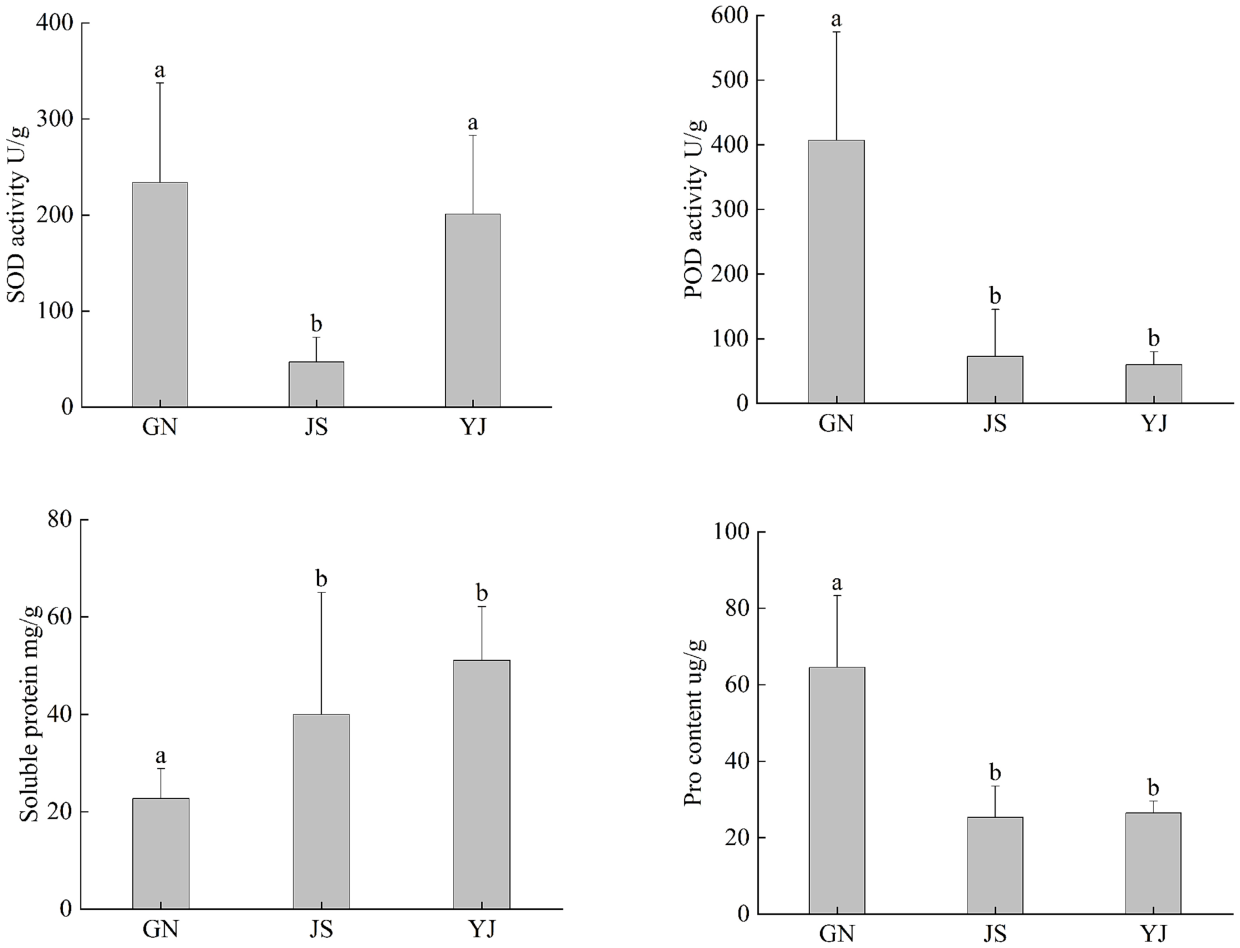
Antioxidant content of *Pinus yunnanensis* leaves in different rainfall areas. The different lowercase letters in the figure indicate significant differences between different regions (*p* < 0.05).

As shown in Figure 1, the changes in the antioxidant system of *Pinus yunnanensis* leaves vary in different regions. There was no significant difference in SOD activity between GN and YJ (*p* > 0.05), while JS showed significant differences between GN and YJ (*p* < 0.05). The POD activity, SP, and Pro content of GN were significantly different from those of JS and YJ (*p* < 0.05), while there was no significant difference in JS and YJ (*p* > 0.05). Both SOD activity and Pro content showed a decreasing and then increasing trend with decreasing rainfall. Compared with JS, the SOD activity in GN and YJ regions increased by 397.66 and 327.49%, respectively. POD activity decreased with decreasing rainfall, while SP content, in contrast, increased with decreasing rainfall. Overall, SOD, POD, and Pro in the GN region have increased compared to the other two areas, while SP content is highest in YJ.

### 3.2 Diversity analysis of bacteria and fungi in different rainfall areas

**Figure 2:**
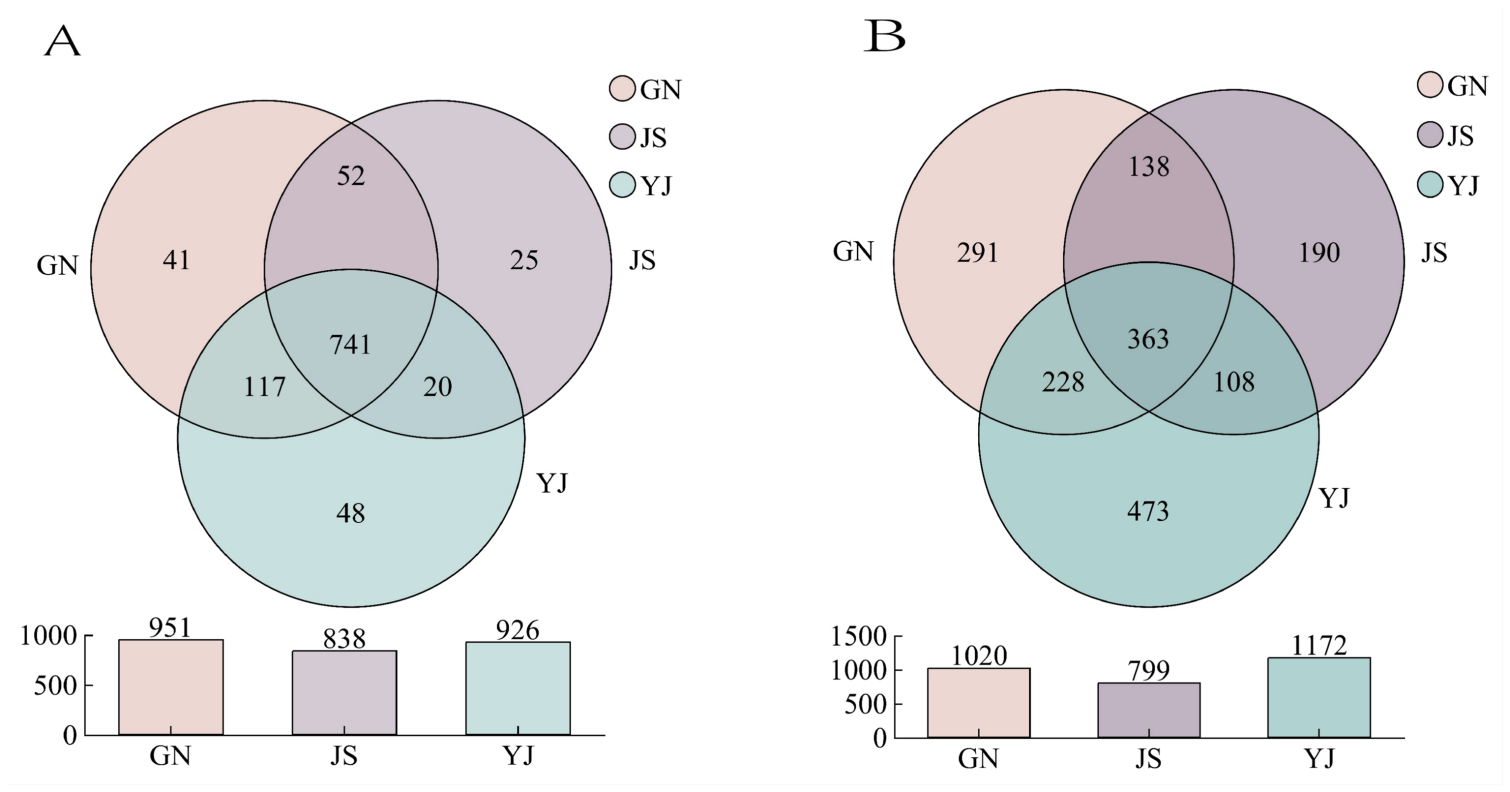
Number of OTUs in soil bacterial **(A)** and fungal **(B)** communities in different rainfall areas.

According to Figure 2A, 951, 838, and 926 soil bacterial OTUs were detected in the natural forest of *Pinus yunnanensis* in GN, JS, and YJ regions, respectively. Among them, the unique OTU numbers are 41, 25, and 48, accounting for 4.31, 2.98, and 5.18%, respectively. The number of bacterial OTUs shared by the three regions was 741, and the GN region has the highest number of shared bacteria, while the JS region has the lowest. Among all the regions, the number of YJ-specific OTUs was the highest, suggesting that YJ had the most significant differences in OTUs from the other regions. On the contrary, JS had the lowest number of unique OTUs, indicating that the region was highly similar to GN and YJ. As shown in Figure 2B, the number of OTUs of soil fungal communities in different rainfall areas were GN: 1020, JS:799, and YJ: 1172, respectively. The number of OTUs unique to GN, JS, and YJ were 291, 190, and 473, with a proportion of 28.53, 23.78 and 40.36%, respectively. The number of shared fungal OTUs was 363, and the number of shared fungal OTUs was lower than the number of shared bacterial OTUs (741) in each region, indicating that different rainfall amounts had a more significant effect on the number of soil fungal OTUs than bacteria in each region.

Alpha diversity can effectively reflect the abundance and diversity of microbial communities (Willis, 2019). In research, Chao1 and ACE indices are often used to evaluate species richness. In contrast, Shannon and Simpson indices are used to measure species evenness and systematically analyze soil microbial community diversity. According to Table 3, the soil’s coverage of bacteria and fungi is above 99.99%, indicating that this sequencing method is reasonable and can reflect the actual situation of soil bacterial and fungal communities under changes in rainfall. The diversity indices (Simpson and Shannon indices) of bacterial and fungal communities in *Pinus yunnanensis* forests showed no significant differences among regions (*p* > 0.05). In contrast, the richness indices (Chao1 and ACE index) showed significant differences among regions (*p* < 0.05). The richness indices (Chao1 and ACE index) and Shannon index of soil bacterial communities were both greatest in GN, the region with the highest precipitation, and lowest in JS, where Chao1 and ACE indices were reduced by 16.98 and 15.75%, respectively, and Shannon index was reduced by 4.46% from GN. For fungi, soil fungi Chao1 and ACE indices were significantly higher in YJ than in GN and JS, and their indices were lowest in JS. YJ showed an increase of 30.83 and 29.19% in Chao1 and ACE indices, respectively, compared to JS; however, both Simpson’s and Shannon’s indices were higher in JS than in other regions. This indicates that soil bacterial diversity and richness were highest in GN, and soil fungal richness was highest in YJ.

**Table 3:** Soil microbial diversity index in different rainfall regions.

Microorganism	Diversity index	GN	JS	YJ
Bacteria	Simpson	0.98 ± 0.003 a	0.98 ± 0.01 a	0.99 ± 0.002 a
	Shannon	5.16 ± 0.16 a	4.93 ± 0.12 a	5.02 ± 0.13 a
	Chao1	865.47 ± 19.85 a	718.48 ± 38.26 b	815.38 ± 17.86 a
	ACE	861.40 ± 21.43 a	725.70 ± 34.87 c	811.17 ± 13.22 b
	Coverage	0.99 ± 0.0006 a	0.99 ± 0.0007 a	0.99 ± 0.001 a
Fungi	Simpson	0.57 ± 0.22 a	0.90 ± 0.09 a	0.80 ± 0.23 a
	Shannon	2.09 ± 0.91 a	3.39 ± 0.71 a	3.09 ± 1.18 a
	Chao1	650.01 ± 102.90 ab	527.25 ± 61.77 b	689.86 ± 44.98 a
	ACE	639.30 ± 96.28 ab	529.59 ± 61.14 b	684.16 ± 44.87 a
	Coverage	0.99 ± 0.002 a	0.99 ± 0.0005 a	0.99 ± 0.0008 a

Different lowercase letters in the table indicate significant differences in diversity index among different regions (*p* < 0.05).

### 3.3 PCoA analysis of soil bacterial and fungal communities

**Figure 3:**
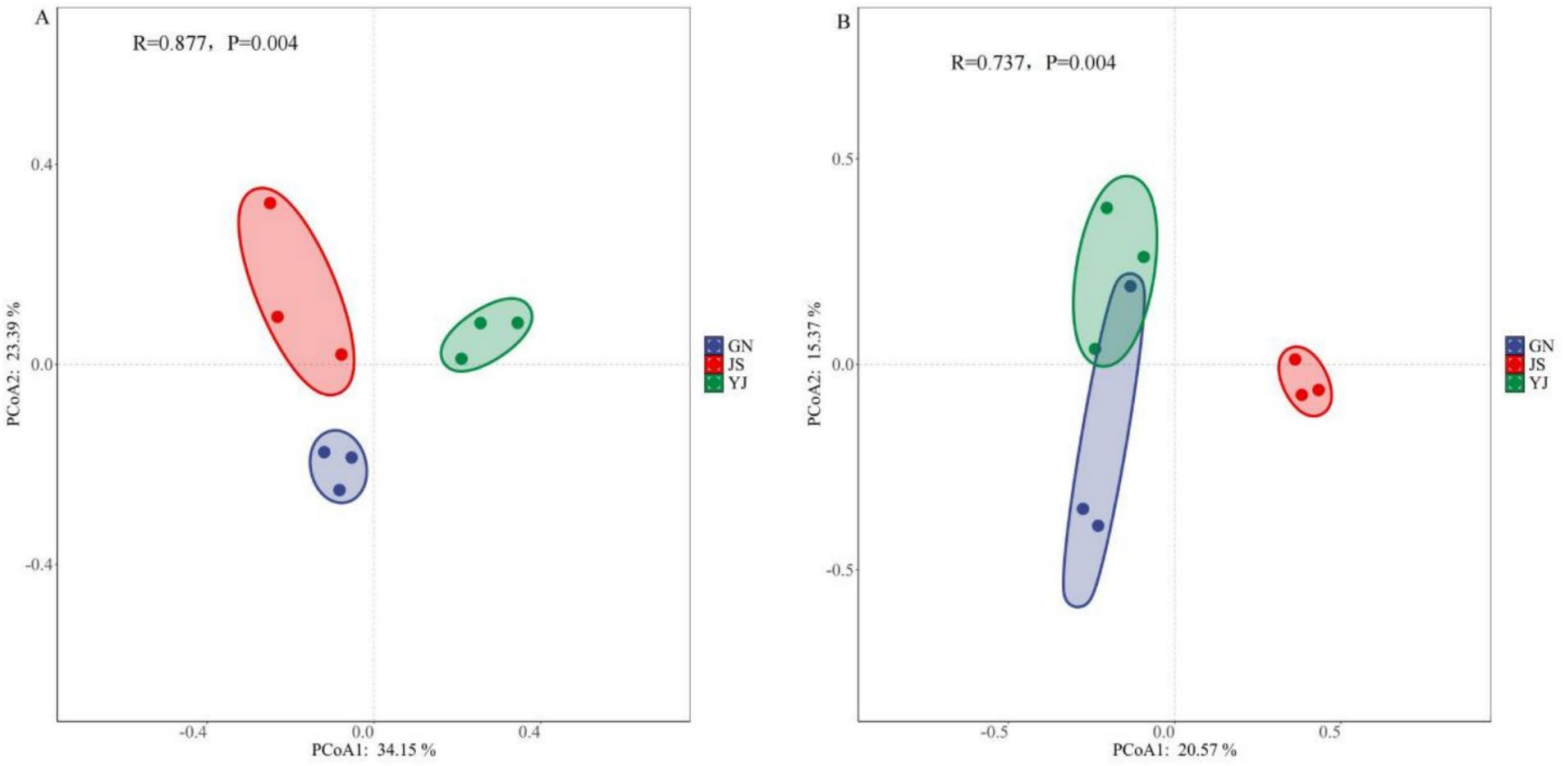
PCoA analysis of soil bacteria **(A)** and fungi **(B)** in different rainfall areas.

Principal coordinate analysis (PCoA) using the Bray–Curtis distance algorithm was used to reveal the effects of rainfall changes on the community structure of soil bacteria and fungi, and an ANOSIM significance test was performed to test the differences in community structure between groups. The results showed that the soil bacterial communities in the three regions of GN, JS, and YJ did not overlap in two-dimensional space, and their distribution locations were relatively dispersed. This suggests that the differences in the structure of soil bacterial communities were more pronounced among the regions (Figure 3A). The soil fungal communities of GN and YJ were more aggregated, indicating the similarity of fungal community structure in GN and YJ. However, the soil fungal community of JS was more separated from the other two areas, indicating that it was more different from the other two areas (Figure 3B). Overall, there were significant differences in the community structure of *Pinus yunnanensis* soil bacteria (*R* = 0.877, *p* < 0.01) and fungi (*R* = 0.737, *p* < 0.01) among the three regions.

### 3.4 Composition of bacterial and fungal communities in *Pinus yunnanensis* soil under different rainfall levels

**Figure 4:**
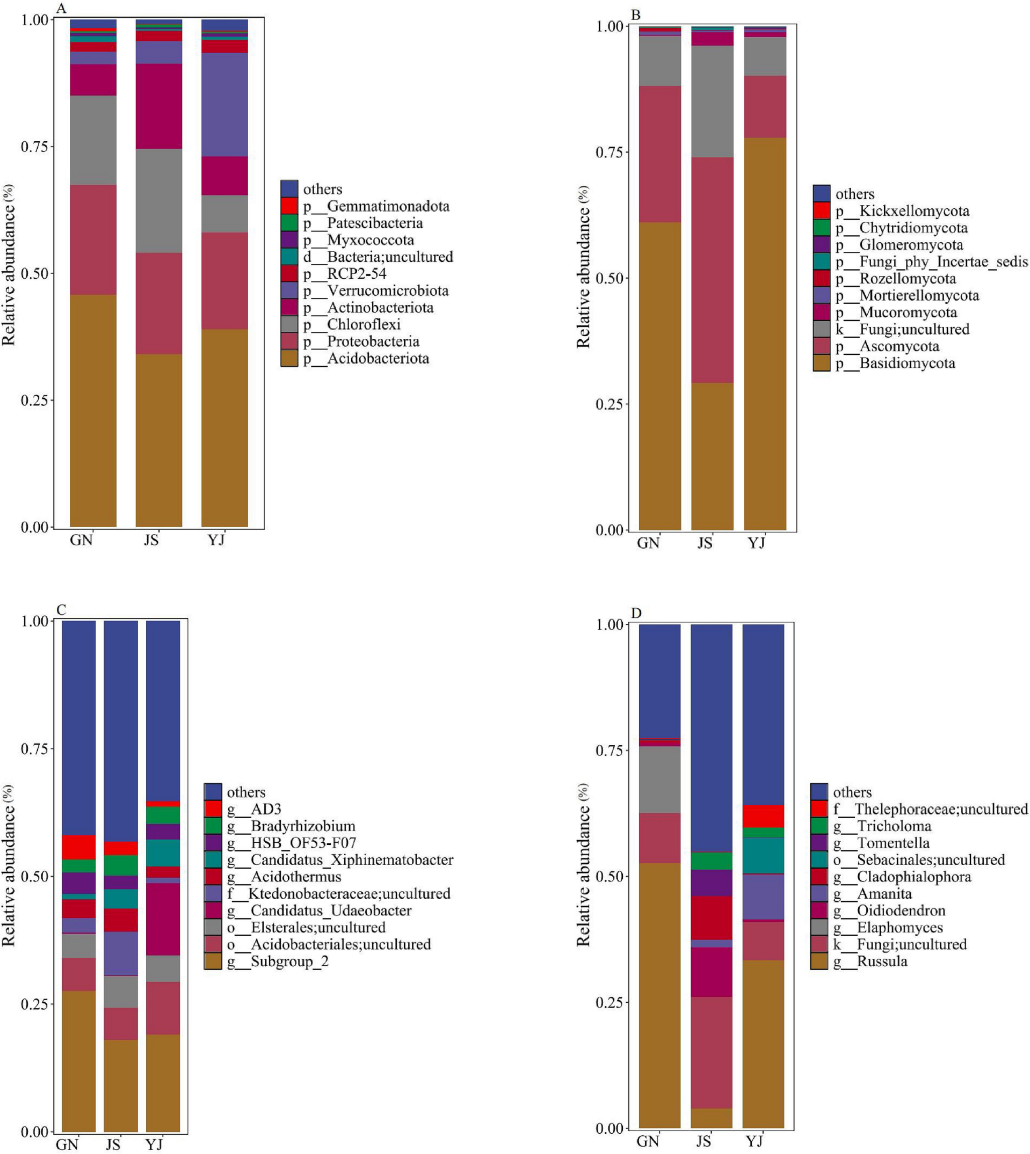
Relative abundance of soil microbial communities in different rainfall areas. Relative abundance of bacterial and fungal communities at the phylum level in **(A,B)**; Relative abundance of bacterial and fungal communities at the genus level in **(C,D)**.

Classify and annotate the obtained bacterial OTU sequences with soil bacteria belonging to 27 phylum, 57 classes, 117 orders, 164 families, 222 genera, and 243 species in three regions. The composition of the top 10 species in terms of abundance at the phylum and genus levels of soil bacteria and fungi is shown in Figure 4. Changes in rainfall affected the relative abundance proportions of soil bacteria and fungi at both phylum and genus taxonomic levels. The primary dominant groups of soil bacteria at the phylum level (Figure. 4A) for GN and JS were as follows: GN: *Acidobacteria* (45.84%), *Proteobacteria* (21.57%), and *Chloroflex*i (17.67%); JS: *Acidobacteria* (34.08%), *Chloroflexi* (20.58%), and *Proteobacteria* (19.89%). Moreover, the main bacterial dominant phyla in YJ were *Acidobacteria* (30.97%), *Verrucomicrobiota* (20.41%), and *Proteobacteria* (19.17%). These dominant bacterial phylum account for over 74% of the total soil bacterial community. At the genus level, the community is mainly composed of the unnamed genera *Subgroup_2* (17.98% ~ 27.56%), *Acidobacteriales;uncultured* (6.26% ~ 10.34%), and *Elsterales*;*uncultured* (4.80% ~ 6.34%) (Figure 4C). The abundance of the unnamed *Subgroup_2* genus in the phylum *Acidobacteria* is highest in GN. *Acidobacteriales;uncultured* had the highest abundance in YJ and the lowest in JS, while *Elsterales*;*uncultured* had the highest abundance in JS, followed by YJ and the lowest in GN.

The statistical analysis of soil fungal communities shows that the soil fungi in the three regions belong to 14 phyla, 55 classes, 134 orders, 277 families, 489 genera, and 646 species. The phylum levels were all dominated by *Basidiomycota* (29.15–77.84%) and *Ascomycota* (12.35–44.87%) as the major fungal phyla (Figure 4B), and these two dominant phyla accounted for more than 74% of the total. Among the three regions, except for JS, which has *Ascomycota* as the first dominant phylum, GN and YJ have *Basidiomycota* as the first and *Ascomycota* as the second dominant phylum. The relative abundance of *Basidiomycota* in GN and YJ is more than twice that of JS. The genus levels of *Russula* (33.33% ~ 52.64%), *Fungi*; U*ncultured* (7.68% ~ 22.15%), and *Elaphomyces* (0.04% ~ 13.32%) are the main communities (Figure 4D). Among them, the relative abundance of *Russula* in the GN and YJ regions is higher than that in the JS region, with an increase of 48.72 and 29.41%, respectively. However, *Elaphomyce*s only exist in large quantities in GN, and their relative abundance in JS and YJ is only 0.06 and 0.04%, respectively.

**Figure 5:**
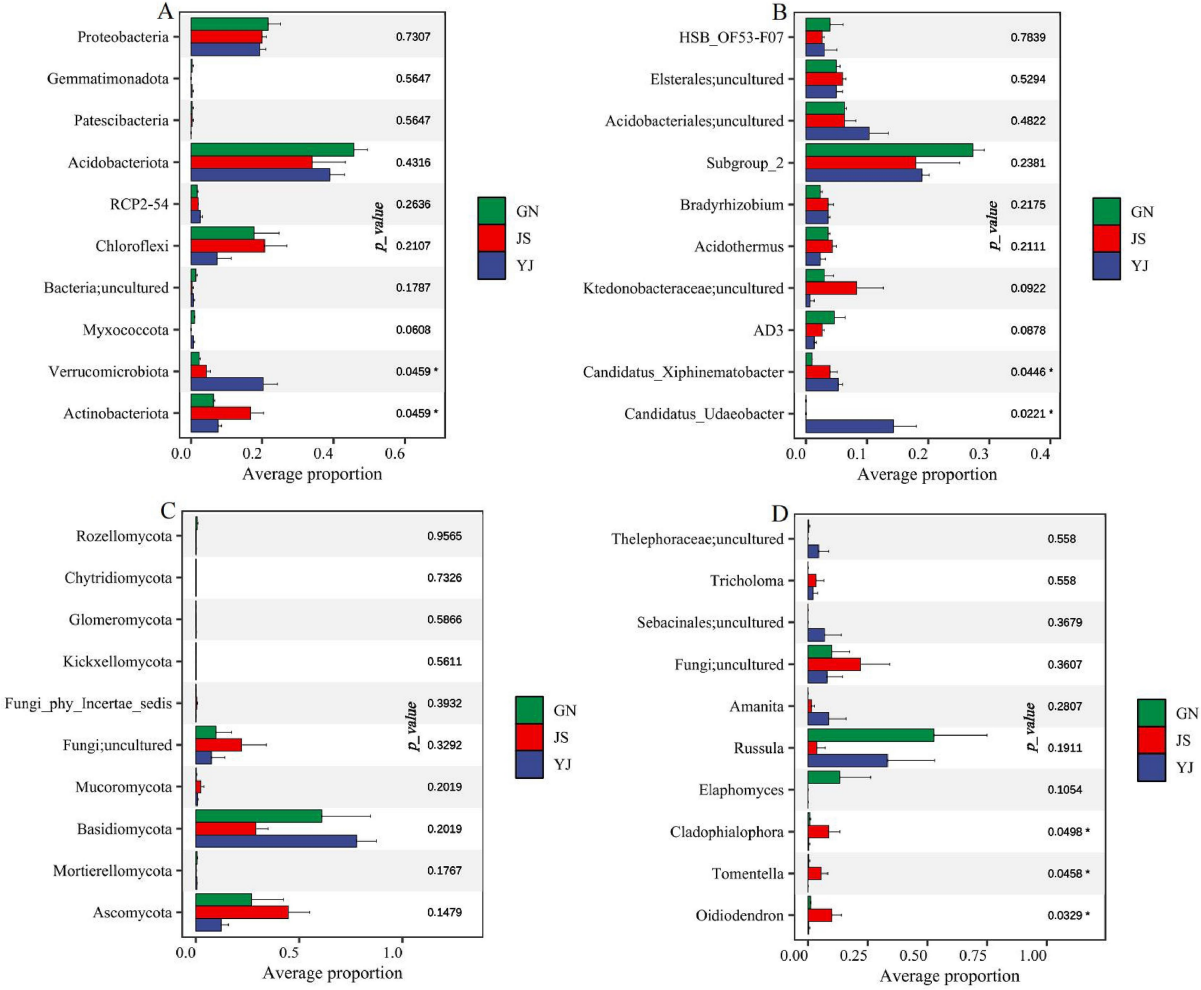
Differences in the abundance of soil microbial communities in different rainfall areas. Differences in bacterial and fungal abundance at the phylum level in **(A,C)**; differences in bacterial and fungal abundance at the genera level in **(B,D)**. *, **, and *** represent *p* < 0 05, *p* < 0.01, and *p* < 0.001, respectively, the same below.

Kruskal–Wallis analysis was conducted on the top 10 bacteria and fungi with relative abundance at the phylum and genus levels to further explore the impact of rainfall changes on soil bacterial and fungal communities. It was found that there were significant differences in bacterial community composition at the phylum and genus levels among the three regions. At the phylum level (Figure 5A), the soil bacterial communities corresponding to different regions showed significant differences (*p* < 0.05) between *Verrucomicrobio*ta and *Actinobacteriota*. While at the genus level (Figure 5B), the difference was significant (*p* < 0.05) on the uncultured bacterial genera *Candidatus_Udaeobacter* and *Candidatus_Xiphinematobacter* in the phylum *Verrucomicrobia*. Whereas none of the fungal community compositions were significantly different at the phylum level (Figure. 5C), *Cladophialophora*, *Tomentella*, and *Oidiodendron* differed significantly at the genus level (*p* < 0.05) (Figure 5D).

### 3.5 Correlation analysis between soil microbial community and antioxidants

**Figure 6:**
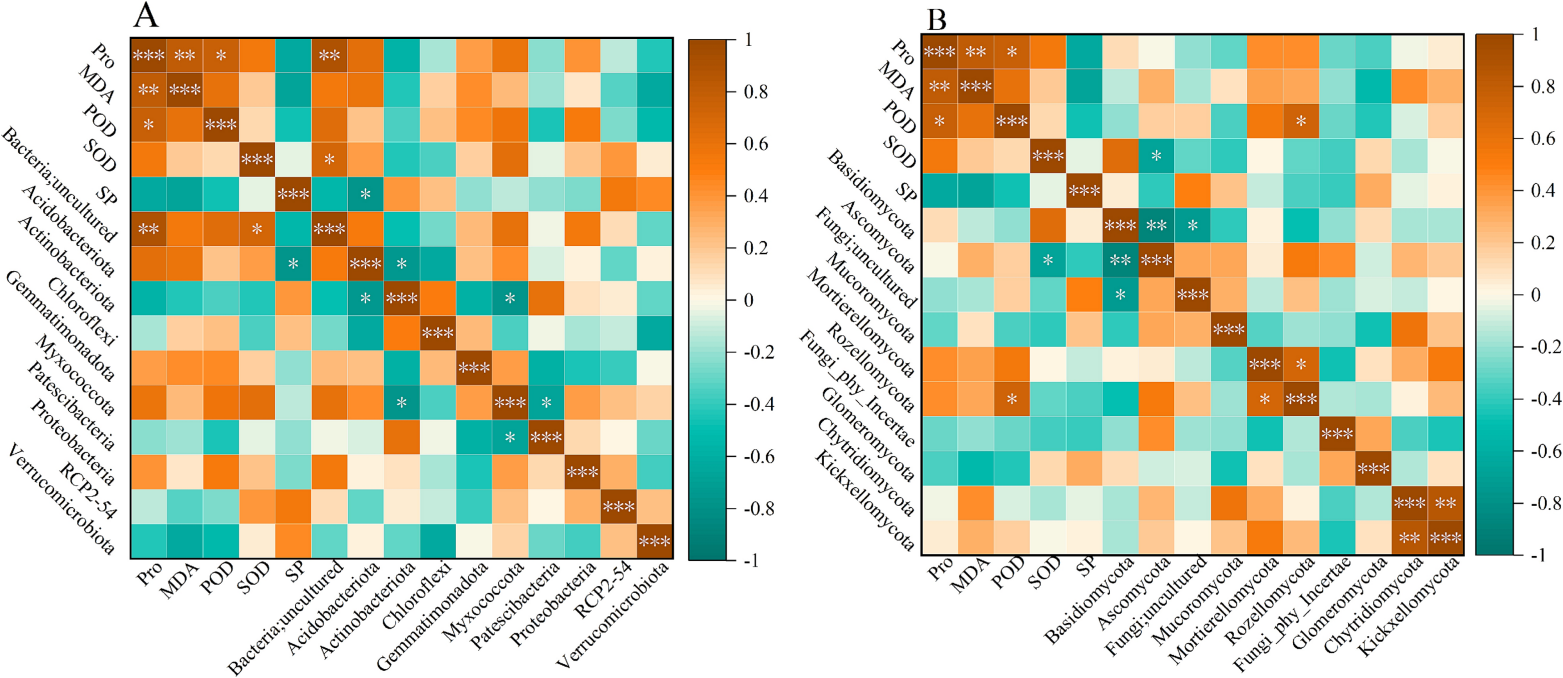
Correlation analysis between antioxidants and major bacterial **(A)** and fungal **(B)** phylum levels.

The correlation analysis between antioxidant enzymes and the dominant phylum of bacteria and fungi is shown in Figure 6. As shown in Figure 6A, *Bacteria;uncultured* was significantly positively correlated with Pro and SOD (*p* < 0.05). The abundance change of *Bacteria;uncultured* promotes the production and accumulation of Pro and SOD, suggesting that this uncultured bacteria may help enhance the plant’s antioxidant and osmotic regulation abilities in coping with adversity and regulating physiological processes. SP was significantly negatively correlated with *Acidobacteriota* (*p* < 0.05). For fungal communities (Figure 6B), POD was significantly positively correlated with *Rozellomycota* (*p* < 0.05), while SOD was significantly negatively correlated with *Ascomycota*. In addition, *Basidiomycota* and *Fungi;uncultured*, and *Ascomycota* were negatively correlated (*p* < 0.05).

### 3.6 Functional analysis of soil bacterial and fungal communities

**Figure 7:**
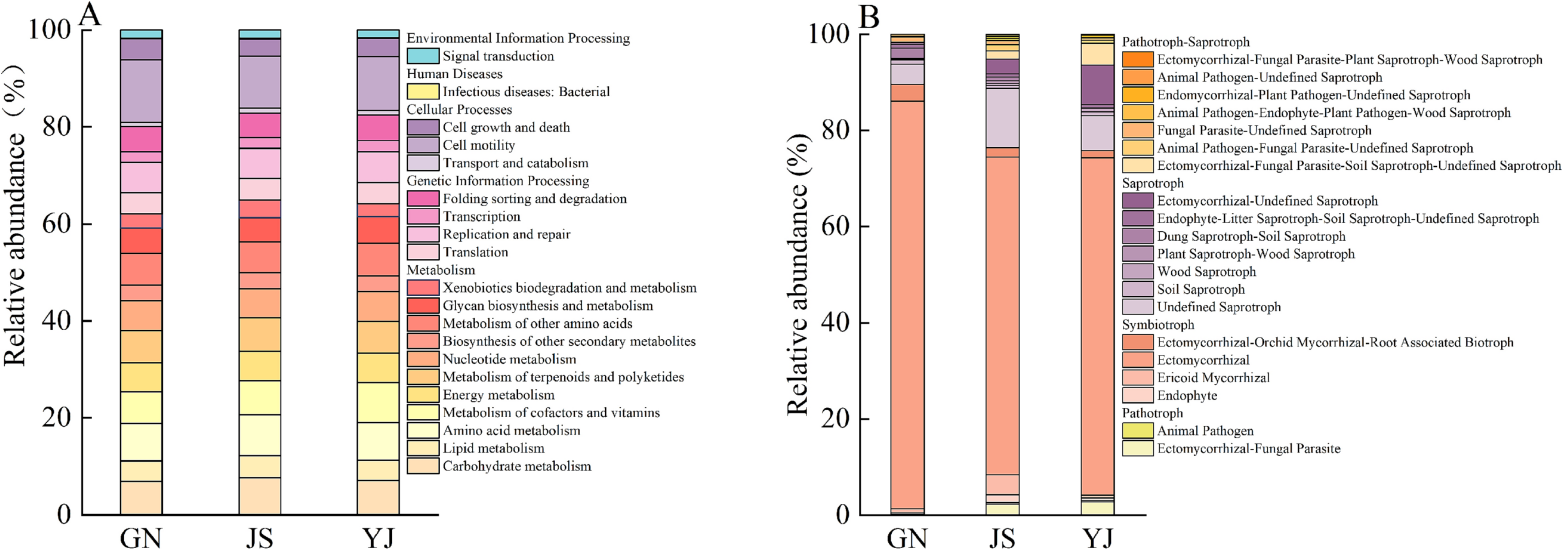
Functional abundance of bacteria **(A)** and fungi **(B)**.

Functional prediction of the soil bacterial community was performed using Tax4Fun with reference to the KEGG Orthology (KO) database. The prediction results showed that all soil samples were annotated with 6 and 30 functional categories on KEGG primary and secondary metabolic pathways, respectively (Figure 7A). The relative abundance of each primary functional pathway in the sample is as follows: metabolism (80.91% ~ 82.34%) > genetic information processing (11.11% ~ 11.94%) > cellular processes (4.30% ~ 4.98%) > environmental information processing (1.90% ~ 1.95%) > human diseases (0.10% ~ 0.11%). Among them, the abundance of metabolism functions in the JS region is higher than in other regions, and the abundance of genetic information processing functions is lower than in other regions. Secondary functional prediction found that soil in the JS region is more expressed in the carbohydrate metabolism and amino acid metabolism pathways. The functions of metabolism of cofactors and vitamins and the metabolism of other amino acids are actively expressed in the YJ region, and GN is most actively expressed in the cell motility pathway.

The FUNGuild functional prediction tool was used to analyze the soil fungal community of *Pinus yunnanensis* ding in terms of nutritional mode, namely *Symbiotroph* (50.84% ~ 89.41%), *Saprotoph* (8.63% ~ 34.81%), *Pathtroph* (0.14% ~ 10.86%), and *Pathotroph Aprotroph* (1.79% ~ 8.58%). Classifying the functional taxa of fungi with more than 1% abundance, *Symbiotroph* mainly consisted of *Ectomycorrhizal*, *Endophyte,* and *Ericoid Mycorrhizal*. Among them, *Ectomycorrhizal* had the highest relative abundance in the GN region and the lowest in the JS region. *Endophyte*, on the other hand, had the highest abundance in the JS region and the lowest in the GN region. *Saprotroph* had the highest abundance in JS, with an increase of 26.18 and 16.98% over GN and YJ, respectively. It was mainly composed of *Undefined Saprotroph*, *Plant Saprotroph-Wood Saprotroph*, *Dung Saprotroph-Soil Saprotroph,* and *Ectomycorrhizal-Undefined Saprotroph. Undefined Saprotroph* has the highest share in JS (32.35%) and the lowest share in GN with only 4.20%.

## 4 Discussion

### 4.1 The impact of rainfall changes on plant antioxidant systems

The interaction between SOD and POD plays an important protective role in plant aging by maintaining the homeostasis of reactive oxygen species, effectively inhibiting membrane lipid peroxidation, and alleviating damage caused by oxidative stress (Liu et al., 2018). In this study, under two completely different precipitation conditions (GN with more rainfall and YJ with less rainfall), the activities of SOD and POD in *Pinus yunnanensis* leaves increased, which is consistent with the response of leaf antioxidant enzymes to rainfall changes in *Artemisia ordosica* Krasch (Wang, 2022). This phenomenon may be due to abundant rainfall causing soil moisture saturation and a decrease in oxygen content in the soil, which forces root systems to initiate anaerobic respiration due to hypoxia. However, the efficiency of anaerobic respiration is low, interfering not only with regular gas exchange in cells but also exacerbating the accumulation of ROS, leading to damage such as membrane lipid peroxidation (Zhang Y. S. et al., 2025). Under drought conditions, plants reduce water loss by closing stomata, but this also limits CO_2_ absorption, leading to a decrease in photosynthetic carbon assimilation capacity. At this point, the amount of light absorbed by plants exceeds the energy consumed by carbon fixation in photosynthesis. This excess light can trigger the production of ROS, such as O_2_^−^ and H_2_O_2_, causing oxidative damage to cells (Wang Z. et al., 2018). SOD and POD are key enzymes in the antioxidant system, therefore, *Pinus yunnanensis* upregulates their enzyme activities to eliminate ROS and maintain cellular homeostasis efficiently. Pro and SP are important osmotic regulators in plants. They regulate cellular osmotic pressure by increasing or decreasing their content when plants are under stress, thereby protecting the plant from damage (Zhong et al., 2018). In this study, both SP and Pro contents were affected by geographical differences, with GN having the highest Pro content and the lowest SP content. It is possible that Pro protects cells from damage by acting as an osmotic agent and free radical scavenger (Li H. Z. 2024) and synergizing with SOD and POD to alleviate oxidative damage. Therefore, *Pinus yunnanensis* may shift resources to osmotic substances such as Pro during adversity while reducing the synthesis of soluble SP. It is also possible that genetic differences could influence the metabolic pathway to favor Pro synthesis in this region. In addition, among the three regions, the JS region has the lowest SOD and POD activity, as well as the lowest Pro content, which may be closely related to the rocky habitat in this region. The soil in rocky areas has poor water and fertilizer retention ability, which limits the growth of plants in stony soil, hinders root development, and reduces the nutrient absorption capacity. The overall growth metabolism may thus weaken, leading to lower levels of SOD, POD, and Pro contents. An on-site investigation found that the JS region has the highest density of *Pinus yunnanensis* seedlings among the three regions. Due to the competition of many plants for limited nutrient resources, individual *Pinus yunnanensis* plants obtain less nitrogen. Furthermore, the total nitrogen content in the region’s soil is the lowest among the three regions, limiting plant access. Since nitrogen is an important component of proteins and enzymes, SOD and POD activities decline significantly under low-nitrogen conditions (Yu et al., 2015).

### 4.2 Impact of rainfall changes on soil microbial diversity and community composition

Soil microbial diversity is a key factor in evaluating the ability of ecosystems to adapt to environmental changes and maintain ecological balance (Li S. F. et al., 2023). Changes in microbial habitat, nutrient cycling, and competition caused by precipitation variation can lead to differences in microbial community diversity, thereby affecting the stability and adaptability of ecosystems. Previous studies have shown that increased precipitation in arid and semi-arid regions can effectively alleviate drought stress and increase microbial abundance, while decreased precipitation further limits the diffusion of the soil matrix and inhibits the growth of soil microorganisms (Gao et al., 2021). In this study, the Shannon and Simpson indices of soil bacterial communities showed no significant differences among different regions, consistent with the findings of Na et al. (2019). However, the Shannon, Chao1, and ACE indices of soil bacteria in the GN region were higher than those in JS and YJ, which may be related to the increase in the relative abundance of soil bacteria with the increase in SOM content (Yao et al., 2024). The humid climate of GN may promote plant growth and increase litter input, thereby increasing soil organic matter and providing more energy and substrates for microorganisms. Conversely, the drier, hotter climates of JS and YJ may lead to faster decomposition and less accumulation of organic matter. Notably, the soil bacterial diversity index in the GN region was higher than that of the YJ region. Whereas, the fungal community diversity in the YJ region was higher than in the GN region. The reason may be that a specific type of bacteria in the soil microbial community has a competitive exclusion effect (Yang et al., 2019). Elevated bacterial diversity can directly or indirectly suppress fungal growth, potentially explaining the lower bacterial abundance and higher fungal diversity observed in YJ.

Changes in precipitation can cause the local extinction of certain OTUs or alter the abundance of bacteria and fungi, thereby altering community composition and promoting the growth of a particular group (Clark et al., 2009). Through the study of species composition, it was found that the dominant phyla of soil bacteria in the GN and YJ regions are *Acidobacteriota*, P*roteobacteria*, and *Chloroflexi*, while the dominant phyla in YJ are *Acidobacteriota*, *Verrucomicrobiota*, and *Proteobacteria*. In this study, the relative abundance of *Acidobacteriota* decreased with decreasing rainfall. This contradicts the findings of Wang et al. (2016), who reported a positive correlation between *Acidobacteriota* abundance and precipitation. Notably, *Acidobacteriota* abundance in YJ (the driest region) was higher than in JS. This anomaly may result from the interaction of environmental factors. A study has found that *Acidobacteriota* in soil prefers acidic environments and fertile soils (Lu et al., 2010). Although the pH values in the three regions of this study were all in the acidic range of 4.88–5.35, the pH value in the YJ region (5.35) was higher than that in the JS region (4.88). Therefore, soil pH was not the primary driver of YJ’s *Acidobacteriota* advantage. YJ is located in a dry, hot valley with significant high-temperature characteristics. Li Y. J. et al. (2023) pointed out that an increase in temperature can accelerate the decomposition process of organic matter by soil microorganisms, release more CO_2_, and increase the abundance of *Acidobacteria*, resulting in a phenomenon where the abundance of *Acidobacteria* in the YJ region is higher than that in the JS region. *Proteobacteria* can decompose organic matter, improve soil fertility, and promote plant growth and development (Xuan et al., 2012). In this study, the relative abundance of *Proteobacteria* decreases with decreasing rainfall, which is consistent with the findings of Sun et al. (2020), who reported that drought significantly reduces *Proteobacteria* abundance in poplar plantations. The reason may be that *Proteobacteria*, as Gram-negative bacteria, have a fast-growing characteristic, and their growth is easily limited by water, which inhibits their abundance under drought conditions (Placella et al., 2012). In addition, *Chloroflexi* is a type of oligotrophic bacteria that mainly participates in CO_2_ fixation and microbial photosynthesis processes (Wang et al., 2024) and is suitable for growth in environments with low nutrient availability (Fierer and Jackson, 2006). In this study, the abundance of *Chloroflexi* was significantly higher in JS than in GN and YJ. Combining the results of field investigations, the unique rocky habitat in the JS region results in substantially lower soil water and fertilizer retention capacity compared to other areas, forming relatively poor soil conditions that may better meet the growth needs of oligotrophic bacteria. The abundance of *Verrucomicrobiota* in the YJ region has shown an “substantial proliferation,” which may result from the combined effects of drought on soil nitrogen cycling processes and *Verrucomicrobiota* itself. On the one hand, previous studies have shown that arid environments can inhibit the activity of enzymes involved in nitrogen cycling and transformation in soil (Steinweg et al., 2012). The climate in the YJ region is relatively dry, which weakens the mineralization process of nitrogen elements in soil and leads to a decrease in the available nitrogen element content in soil (Sun et al., 2017). On the other hand, relevant studies have shown that *Verrucomicrobiota* is sensitive to nitrogen content, and its abundance is negatively correlated with soil nitrogen content (Zhang et al., 2018). The low-nitrogen environment caused by drought in the YJ region has created suitable living conditions for *Verrucomicrobiota*, giving it a competitive advantage and increasing its abundance. In addition, the relative abundance of *Chloroflexi* in YJ is the lowest among the three regions, suggesting a correlation with the explosive growth of *Verrucomicrobiota* abundance. From the perspective of niche theory and microbial competition, microorganisms compete for nutrients such as carbon and nitrogen sources in ecosystems, and the two may overlap in resource utilization and ecological functions, which requires further investigation.

For fungi, *Basidiomycota* and *Ascomycota* are the two dominant phyla in *Pinus yunnanensis* forest soils, consistent with findings in plants such as *Dendrocalamus brandisii* (Zhu et al., 2023) and *Quercus mongolica* (Guo et al., 2022). Both *Basidiomycota* and *Ascomycota* can promote soil carbon cycling, making them more suitable for environments with low soil moisture content and good aeration conditions (Xu et al., 2022). In this study, the relative abundance of *Basidiomycota* was highest in YJ and lowest in JS. This pattern may be related to differences in soil organic carbon (SOC) content, as YJ had the highest SOC among the regions, providing ample carbon source for *Basidiomycota*, which may contain recalcitrant organic compounds such as lignin and cellulose. Because *Basidiomycota* secretes extracellular oxidases and hydrolases to break down complex compounds such as lignin (Sergentani et al., 2016), it has a competitive advantage in YJ’s high-carbon environment. In addition, *Basidiomycota* can also symbiotically form mycorrhizae with plants, thereby enhancing vegetation stress resistance (Tang et al., 2015). In the typical dry-hot valley area of YJ, plants tend to form a symbiotic relationship with *Basidiomycota* to enhance their stress resistance in response to water scarcity pressure. Establishing this symbiotic relationship provides *Basidiomycota* with a stable carbon source and living space, while the plant’s demand for mycorrhizal fungi also promotes the massive reproduction of *Basidiomycota*, thereby increasing its relative abundance in the soil. However, the *Pinus yunnanensis* of GN and JS have a relatively humid environment and a lower dependence on *Basidiomycota*, which limits their growth. This also confirms the claim that *Basidiomycota* is more suitable for low soil moisture content survival. Another reason *Basidiomycota* has the lowest abundance in JS may be the lower soil nutrient status in the region. When soil nutrients decrease, *Basidiomycota* will gradually be replaced by *Ascomycota*, leading to a decrease in the proportion of *Basidiomycota* (Sterkenburg et al., 2015). Therefore, the relative abundance of *Basidiomycota* is the lowest in the JS region. *Ascomycota* is a saprophytic or symbiotic bacteria that decomposes difficult-to-degrade organic matter and plant and animal residues, increasing the soil’s organic matter content. In this study, the relative abundance of *Ascomycota* is highest in the JS region and lowest in the YJ region. Although the GN region has abundant rainfall, which can promote the decomposition of surface litter (Feng, 2024), the sampling season is the rainy season. The continuous high moisture content causes soil pores to be filled with water, resulting in a significant decrease in air permeability. *Ascomycota* prefers soil conditions with good air permeability (Sui et al., 2016), and this hypoxic environment inhibits its metabolic activity and community development. Although the substrate is abundant, forming a dominant microbial community is difficult. Due to the low rainfall, soil moisture becomes a limiting factor for litter in YJ. The dry environment slows down the litter decomposition, resulting in insufficient substrates for *Ascomycota*. This, in turn, limits the growth and reproduction of this type of fungus, making its relative abundance the lowest. In contrast, the soil moisture content in the JS region is moderate, which not only ensures the required humidity for litter decomposition but also maintains good air permeability. This makes it more suitable for the growth and reproduction of *Ascomycota*, ultimately exhibiting the highest relative abundance. At the genus level, *Russula* is a common *Ectomycorrhizal* that plays an important role in the growth and development of *Pinaceae* species (Jiang et al., 2024). The mycelium of *Russula* fungi can infect the root systems of various plants to form ectomycorrhiza and regulate the symbiotic relationship by secreting a large amount of organic matter into the soil surrounding the roots. These secretions and volatile organic compounds promote plant roots’ growth, enhance nutrient uptake, reduce root diseases, and improve plant drought resistance (Li, 2021). In this study, the relative abundance of *Russula* in GN and YJ regions was significantly higher than in JS. This result suggests that GN and YJ *Pinus yunnanensis* forests may have stronger mycorrhizal symbiosis dependence and stress resistance potential. It is worth noting that there is a relatively high proportion of undefined fungal groups in the JS region, which reveals the particularity of the fungal community structure in this area and requires further research.

### 4.3 Functional prediction of soil microbial community

The changes in soil bacterial community structure caused by moisture will further affect the functional composition of soil bacteria. In addition, soil bacterial communities mainly consume carbohydrates, amino acids, and energy through metabolic activities and participate in energy exchange among bacterial communities and between communities and the environment through membrane transport to maintain community functioning (Yang et al., 2020). This study shows that soil bacterial functions are primarily metabolic, with metabolism-related functions accounting for over 80% of all functions. Among them, the abundance of secondary functions such as amino acid and carbohydrate metabolism in soil bacterial communities in the JS region was slightly higher than in GN and YJ. However, the higher metabolic function of bacteria did not promote optimal growth of *Pinus yunnanensis*, which may relate to insufficient key functional fungi (e.g., ectomycorrhizal fungi) in this region. As a key link in plant–soil interactions, *ectomycorrhizal* fungi play an irreplaceable role in plant nutrient acquisition and growth regulation. Research shows that the mycelium of *Ectomycorrhizal* can replace root hairs to absorb soil moisture below 30 cm, expanding the absorption area and range of plant root water, thereby enhancing the host plant’s absorption of difficult-to-move elements and trace elements (Li et al., 2025) and helping plants transform, absorb, and transport various elements such as phosphorus and potassium in the soil (Gao et al., 2009), thereby improving the host’s nutritional status and promoting plant growth (Wang and Ding, 2013). In this study, the relative abundance of *Ectomycorrhizal* fungi in the JS region was less than half that in the GN and YJ regions, severely limiting the positive regulatory effect of the mycorrhizal symbiosis system on plants. Although the metabolic function of soil bacteria in the JS region is relatively active, capable of decomposing organic substrates to produce small-molecule nutrients that plants can absorb, the lack of synergistic effects of *Ectomycorrhizal* fungi makes it difficult for plants to efficiently obtain key nutrients such as N and P in the soil. In addition, the absence of *Ectomycorrhizal* fungi may disrupt the signal transmission network between plants and microorganisms, affecting the plant’s nutrient absorption and regulation mechanisms, leading to an imbalance between metabolic functions and plant growth needs. Therefore, even if the abundance of soil bacterial metabolic functions is high in the JS region, it still cannot compensate for the insufficient impact of the *Ectomycorrhizal* fungi on *Pinus yunnanensis* growth.

Fungi can produce multiple nutritional pathways to adapt to environmental changes, with different substrate preferences and ecological strategies (Yang et al., 2024). *Symbiotrophs* and *Saprotrophs* are the core components of fungal communities in terrestrial ecosystems. *Symbiotrophs* improve the absorption efficiency of soil nutrients and promote plant growth by forming symbiotic relationships with plants. *Saprotrophs*, as important decomposers in soil, play a crucial role in soil nutrient cycling (Peng et al., 2022). In this study, *Symbiotrophs* dominated soil fungal communities in all three regions, consistent with findings by Wen et al. (2024). The symbiotic system formed between the *Pinus yunnanensis* root system and *Ectomycorrhizal* fungi is a key factor leading to a high proportion of *Symbiotrophs*. Studies have shown that the root system of *Pinus yunnanensis* can symbiotically form mycorrhizae with various fungi, such as *Russula*, *Boletus*, etc. (Yu et al., 2007). Diversified mycorrhizal fungi can not only adapt to different soil environments but also improve the stability and productivity of ecosystems through functional complementarity, thereby maintaining the high abundance characteristics of *Symbiotrophs*. This study also found that the relative abundance of *Saprotrophs* was the highest in the JS region, while the abundance of *Ectomycorrhizal* fungi was the lowest. It is speculated that this phenomenon is related to the coexistence and competition between *Saprotrophs* and *Ectomycorrhizal* fungi. *Ectomycorrhizal* fungi, with their well-developed hyphae, can quickly obtain nitrogen from the soil, limiting the activity of *Saprotroph* fungi and inhibiting their activity (Chen H. et al., 2025). This study observed that the abundance of *Ectomycorrhizal* fungi in the JS region is relatively low, effectively alleviating the competition pressure for soil nitrogen. Thus, *Saprotrophs* can obtain more nitrogen resources for their own growth and reproduction, gradually occupying a dominant position in the community. This ultimately leads to a significantly higher relative abundance of *Saprotrophs* than *Ectomycorrhizals* in the JS region. Another study found that when the proportion of *Saprotroph* is higher than that of *Symbiotroph*, the stress resistance of trees continues to weaken, thereby increasing the risk of disease occurrence (Wang S. et al., 2018). In this study, the ratio of *Saprophytic* fungi to *Saprotrophs* fungi is as follows: JS (68.47%), YJ (24.53%), and GN (9.66%), which indirectly explains the poor growth of JS *Pinus yunnanensis*. Research has shown that *Wood Saprotrophs* can form water transport channels through hyphae, enhancing plant absorption of soil moisture (Gilmartin et al., 2022). In this study, except for *undefined Saprotroph*, the relative abundance of *Plant Saprotroph-Wood Saprotroph* in the JS region and *Wood Saprotroph* in the YJ region all show relatively high relative abundance. The *Wood Saprotroph* existing in various regions has established stable water transport channels, ensuring that *Pinus yunnanensis* can obtain a sustained water supply in its natural state and meet its growth needs. This microbial-mediated water regulation mechanism plays an important supporting role in the ecological stability of *Pinus yunnanensis* natural forests without management measures.

### 4.4 Relationship between antioxidant system and microbial community composition

In this study, SP was significantly negatively correlated with *Acidobacteriota* (*p* < 0.05).

Previous studies have shown that *Acidobacteriota* is more active in high-moisture environments (Nguyen et al., 2018). As a key osmotic regulator in plants, SP can effectively improve the stability of cell membranes and enhance their adaptability to drought environments by increasing its content under drought stress (Jin et al., 2024). Moreover, this study’s sampling time was during the rainy season, when the soil moisture content was relatively high. Under such conditions, the activity of *Acidobacteriota* increased. Meanwhile, due to sufficient soil moisture, plants do not need to accumulate excessive SP to regulate osmotic balance and cope with drought, which may decrease SP content. This study found that *Rozellomycota* was significantly and positively correlated with POD (*p* < 0.05). *Rozellomycota* tends to exist in hypoxic environments (Hans-Peter et al., 2016), and it exhibits strong adaptability to extreme soil environments. However, hypoxic environments inhibit aerobic respiration in plants, leading to insufficient ATP synthesis and hindering processes such as plant substance synthesis and nutrient absorption. Additionally, secondary metabolites such as alcohol produced by anaerobic respiration can be toxic to plants (Liu et al., 2025). Under these conditions, plants experience a metabolic imbalance of ROS, leading to the accumulation of peroxides such as H₂O₂. In response, plants activate ROS-scavenging enzyme systems to enhance the activity of antioxidant enzymes such as POD, clearing excess ROS, delaying plant aging, and maintaining normal plant growth and development (Ma et al., 2025). Therefore, in hypoxic environments enriched with Rozellomycota, plants elevate POD activity to counteract oxidative stress, explaining the observed positive correlation. SOD was significantly negatively correlated with *Ascomycota* (*p* < 0.05). This may occur because *Ascomycota* is a saprophytic bacterium that plays an important role in decomposing residues and organic matter (Chen et al., 2021). During the rainy season, increased rainfall accelerates litter decomposition, reducing undecomposed plant residues on the soil surface. This leads to a corresponding decrease in the nutrient substrates provided for *Ascomycota*, resulting in a reduction in their abundance. In addition, studies have shown that excessive precipitation can inhibit soil respiration (Wang et al., 2019), while anaerobic conditions impair root function, hindering plant development (Xu et al., 2025). At this time, the production of ROS in plant cells increases. To maintain intracellular redox balance, plants depend on antioxidant enzyme systems such as SOD to scavenge excess ROS and mitigate oxidative damage.

## 5 Conclusion

This study compared soil microbial communities and plant antioxidant enzyme systems in *Pinus yunnanensis* forests in GN, JS, and YJ regions, revealing the mechanisms by which regional ecological differences affect soil microbial–plant relationships. The main conclusions are as follows: In terms of soil microbial community diversity and structure, the Shannon, Chao1, and ACE indices of soil bacterial communities were highest in the GN region. The YJ region had the highest Chao1 and ACE indices for soil fungal communities. Bacterial community structure differed significantly among regions. *Acidobacteria*, *Proteobacteria*, and *Chloroflexi* were the dominant phyla in GN and JS, whereas *Acidobacteria*, *Verrucomicrobiota*, and *Proteobacteria* dominated YJ. Although *Basidiomycota* and *Ascomycota* were dominant in all three regions (accounting for ≥74%), fungal community structures were highly similar between GN and YJ. Correlation analysis between soil microbial community and plant antioxidant enzymes shows that the Pro content and SOD activity of *Pinus yunnanensis* leaves were significantly and positively correlated with *Bacteria*;*uncultured* (*p* < 0.05). The SOD activity and SP content were significantly negatively correlated with *Ascomycota* (*p* < 0.05), indicating the presence of *Bacteria*; U*ncultured,* which may be involved in plant stress regulation, while *Ascomycota* ecological function may have an antagonistic relationship with plant stress resistance. At the functional level of microbial communities, soil bacteria in the JS region are most active in metabolism pathways, followed by the YJ region; the GN region exhibits unique functional advantages in the cell motility pathway. The soil fungal community is mainly composed of *Symbiotroph* and *Saprotroph* functional groups, and rainfall changes significantly impact the functional abundance of fungal communities more than that of bacterial communities. In summary, the soil under the *Pinus yunnanensis* forest in the GN area had high bacterial community diversity and richness and contained bacterial groups closely related to plant growth. At the same time, *Symbiotroph* fungi account for the highest proportion. Combined with field investigation data, it has been confirmed that the growth status of *Pinus yunnanensis* in the GN area is significantly better than that in the JS and YJ areas, indicating that a healthy soil microbial community structure, especially a high proportion of symbiotic functional bacteria, plays a key role in promoting plant growth and development.

## Data Availability

The original contributions presented in the study are publicly available. This data can be found here: deposited in the NCBI Short Read Archive (SRA), accession numbers PRJNA1272485.

## Footnotes

^1^ https://www.cloudtutu.com/

## Appendix

**Table A1:** Abbreviations for *Pinus yunnanensis* sampling sites, soil, and antioxidant systems.

Full name	Abbreviation
Sampling site	
Guangnan County, Yunnan Province, China	GN
Jianshui County, Yunnan Province, China	JS
Yuanjiang County, Yunnan Province, China	YJ
Soil	
Soil pH	Soil pH
Total soil nitrogen	TN
Total soil phosphorus	TP
Soil organic carbon	SOC
Antioxidant system	
Superoxide Dismutase	SOD
Peroxidase	POD
Proline	Pro
Soluble Protein	SP
Principal coordinate analysis	PCoA
Alpha diversity	α-diversity
Reactive oxygen species	ROS
